# Review of Integrated Management of Childhood Illness (IMCI) in 16 countries in Central Asia and Europe: implications for primary healthcare in the era of universal health coverage

**DOI:** 10.1136/archdischild-2019-317072

**Published:** 2019-09-26

**Authors:** Susanne Carai, Aigul Kuttumuratova, Larisa Boderscova, Henrik Khachatryan, Ivan Lejnev, Kubanychbek Monolbaev, Sami Uka, Martin Weber

**Affiliations:** 1 University Witten Herdecke Faculty of Medicine, Witten, Germany; 2 World Health Organization Regional Office for Europe, Copenhagen, Denmark; 3 WHO CO Moldova, World Health Organization Regional Office for Europe, Chisinau, Moldova; 4 WHO CO Armenia, World Health Organization Regional Office for Europe, Yerevan, Armenia; 5 WHO CO Kyrgyzstan, World Health Organization Regional Office for Europe, Bishkek, Kyrgyzstan; 6 WHO Office Pristina, World Health Organization Regional Office for Europe, Copenhagen, Denmark

**Keywords:** paediatric practice, health service, general paediatrics, qualitative research, children's rights

## Abstract

The Integrated Management of Childhood Illness (IMCI) was introduced in Central Asia and Europe to address the absence of evidence-based guidelines, antibiotics misuse, polypharmacy and overhospitalisation. This study in 16 countries analyses status, strengths of and barriers to IMCI implementation and investigates how health systems affect the problems IMCI aims to address. 220 key informants were interviewed ranging from 5 to 37 per country (median 12). Data were analysed for arising themes and peer-reviewed. IMCI has not been fully used either as a strategy or as an algorithmic diagnostic and treatment decision tool. Inherent incentives include: economic factors taking precedence over evidence and the best interest of the child in treatment decisions; financing mechanisms and payment schemes incentivising unnecessary or prolonged hospitalisation; prescription of drugs other than IMCI drugs for revenue generation or because believed superior by doctors or parents; parents’ perception that the quality of care at the primary healthcare level is poor; preference for invasive treatment and medicalised care. Despite the long-standing recognition that supportive health systems are a requirement for IMCI implementation, efforts to address health system barriers have been limited. Making healthcare truly universal for children will require a shift towards health systems designed around and for children and away from systems centred on providers’ needs and parents’ expectations. Prerequisites will be sufficient remuneration, sound training, improved health literacy among parents, conducive laws and regulations and reimbursement systems with adequate checks and balances to ensure the best possible care.

## Introduction

Child mortality reportedly halved from 12 million to <6 million deaths globally during the Millennium Development Goals period from 1990 to 2015.^1^ The global strategy of the *Integrated Management of Childhood Illness* (IMCI), launched by WHO and UNICEF in 1995, to end preventable child mortality may be partially credited for this success.^2^ The strategy was devised as a three-pronged approach: (1) improving health worker performance at primary healthcare (PHC) level, which was subsequently expanded to the referral level, (2) strengthening health system performance and (3) enhancing community and family practices.^3^ IMCI was originally intended for implementation in high-mortality and predominantly low-income settings (with an under-5 mortality rate of >40/1000), with the primary aim of reducing mortality. However, its principles also address the promotion of child health and development, quality of care improvement including efficient, standardised care for common illnesses and the reduction of childhood morbidity. In the WHO European region, IMCI was introduced in the late 1990s in 15 of its member states and the territory of Kosovo (namely: Albania, Armenia, Azerbaijan, Belarus, Georgia, Kazakhstan, Kyrgyzstan, Moldova, Romania, Russia, Tajikistan, Turkey, Turkmenistan, Ukraine, Uzbekistan and the territory of Kosovo) (in accordance with UNSCR 1244 (1999); for the purpose of this article countries include countries and territories). While huge disparities in childhood mortality existed (and still persist) in the region, high mortality was not the main concern in many countries that considered implementing IMCI. In the European region, poor quality of care, absence of evidence-based guidelines, the misuse of antibiotics, polypharmacy and overhospitalisation warranted the implementation of a system like IMCI.^4^ Health system inefficiencies were common. Twelve of the 16 countries have been transitioning from the centralised Semahsko health systems of the Soviet Union focusing on secondary and tertiary care to decentralised models. Primary healthcare was expanded by retraining obstetrician-gynaecologists and paediatricians to become general practitioners or family doctors providing care to all including children.^5^ These 10-month retraining efforts included training on the IMCI treatment algorithms. In 2016, WHO, UNICEF, the United States Agency for International Development and the Gates Foundation carried out a strategic review of IMCI at a global level, to identify options for future development of child health guidelines, building on the lessons learnt.^6^ To complement the Global review, the WHO Regional Office commissioned an in-depth review of the status of IMCI implementation in the 16 countries in the WHO European region that considered introducing IMCI or have done so. The main objective of the European IMCI review was to assess the status of IMCI implementation, its relevance and effectiveness in providing quality healthcare to children and to use the findings to inform future strategies to improve child health and well-being in Central Asia and the European region.

In this paper, we present findings of the regional IMCI review. We present the main strengths of and barriers to IMCI implementation, as stated by key informants. We discuss how health and other systems affect the problems that IMCI is trying to address and potential ways forward.

## Methods

Individual interviews and focus group discussions were carried out at national, district and facility level using semi-structured questionnaires followed by an iterative questioning technique and root cause analysis with key informants in 16 countries and territories, namely Albania, Armenia, Azerbaijan, Belarus, Georgia, Kazakhstan, the territory of Kosovo, Kyrgyzstan, Moldova, Romania, Russia, Tajikistan, Turkey, Turkmenistan, Ukraine and Uzbekistan. This qualitative in-depth approach was chosen to facilitate externalising inherent problems that may be overlooked when familiar routines, forms of interaction, power relationships and established interpretations of situations and strategies are taken for granted.

Key informants involved in or exposed to IMCI implementation were identified through formal and informal networks including through ministries of health (MoH) and WHO country offices. In addition, WHO and UNICEF publications and reports were reviewed for potential key informants and the interviewees were asked to indicate further potential key informants.

A total of 220 key informants were interviewed ranging from 5 to 37 per country with a median of 12. An additional four key informants were interviewed but excluded from further analysis, as their respective countries did not go ahead with IMCI implementation.

Key informants included doctors, nurses and in former Soviet Union countries *feldshers* (healthcare professional with clinical responsibilities between those of physicians and nurses) providing care to children, representatives of the MoH and country offices of WHO and UNICEF and other international and non-governmental organisations. Key informants’ profiles can be found in table 1.

**Table 1:** Key informants’ profile

Key informants	
Specialists/doctors working at referral level	56
Doctors working at primary care level	44
Nurses/feldshers	29
Ministry of health staff	32
Staff of international organisations/non-governmental organisations	28
Academia and professional organisations	31
	**220**

Investigators facilitated open-ended discussions on predefined questions and the diagram of the IMCI impact model in search of statements by key informants that could illuminate why IMCI implementation was successful or not in improving child health in the respective country settings. The IMCI impact model is described elsewhere.^7^ Follow-up questions and an iterative interrogative technique were used to elaborate responses in more detail. A methodology outlining this technique was developed prior to the data collection that is primarily based on *participatory action research* by Baum *et al*.^8^ Answers were directly transcribed verbatim or, when consent was granted, audiotaped and then transcribed.

Before the key informant interviews, desk reviews of relevant material were carried out including information collected via a previsit questionnaire completed by the MoH and the WHO country office. Information collected from the previsit questionnaire, desk review, interviews and focus group discussions was triangulated for cross-validation and analysed for commonly arising themes. Transcripts were reviewed to identify the most frequently mentioned strength and weaknesses of the IMCI approach. Verbatim comments by key informants specific to each setting were grouped into themes, for example, *IMCI implementation reduced polypharmacy* by the lead investigator.

These groupings were then reviewed by the respective country investigator and a minimum of one other investigator for accuracy and consistency and agreed on by consensus of the entire group.

### Patient involvement

This is a key informant review carried out at national, district and health facility level and no individual patient data were included.

## Results

The IMCI strategy was introduced in 16 countries in the European region through orientation meetings at national level. Of the 16 countries, 14 had gone ahead with the piloting of IMCI training in 2 to 3 districts (table 2).

**Table 2:** Implementation of IMCI by component in 14 countries of the WHO European region

	Overall	Albania	Armenia	Azerbaijan	Georgia	Kazakhstan	Kosovo*	Kyrgyzstan	Moldova	Russian Federation	Tajikistan	Turkey	Turkmenistan	Ukraine	Uzbekistan
1. Improving health worker performance															
At the primary healthcare level															
Health workers training on IMCI algorithm	**14**	+	+	+	+	+	+	+	+	+	+	+	+	+	+
Follow-up after training	**14**	+	+	+	+	+	+	+	+	+	+	+	+	+	+
At the hospital level															
Introduction of WHO PB	**12**	–	+	–	+	+	+	+	+	+	+	+	+	+	+
Hospital assessment	**11**	–	+	–	+	+	+	+	+	+	+	–	+	+	+
Training on WHO PB	**11**	–	+	–	+	+	+	+	+	+	+	–	+	+	+
PB adopted as national treatment guideline	**6**	–	+	–	–	+	–	+	+	–	+	–	–	–	+
Implementation of improvement activities	**8**	–	+	–	–	+	+	+	+	+	+	–	–	–	+
2. Health systems strengthening															
Inclusion of IMCI drugs in National Essential Drug list	**12**	+	+	+	–	+	+	+	+	+	+	–	+	+	+
IMCI drugs are available free of charge at all times	**4**	–	–	–	–	+	–	+	+	–	–	–	+	–	–
Supportive supervision mechanism	**1**	–	–	–	–	/	–	–	–	–	/	–	–	–	+
Addressed inconsistencies of classification vs ICD 10	**2**	–	–	–	–	+	–	–	–	–	–	–	–	–	+
Addressed policy inconsistencies†	**2**	–	–	–	–	+	–	–	+	–	–	–	–	–	–
3. Community component															
Campaigns	**9**	+	–	+	–	+	–	+	+	–	+	–	+	+	+
Home visits (integration of IMCI messages)	**8**	–	+	–	–	+	+	+	+	–	+	–	+	–	+
EIC materials for parents	**11**	+	+	+	–	+	–	+	+	+	+	–	+	+	+

*In accordance with the United Nations Security Council resolution 1244 (1999).

†For example, sanitary epidemiological services’ requirements for diarrhoea management.

/, aspect reported as partially present; +, aspect reported; −, aspect reported not present; EIC, education, information and communication; ICD, International Classification of Diseases; IMCI, Integrated Management of Childhood Illness; PB, Pocket Book.

Reasons cited for not piloting IMCI included: IMCI not being relevant and too basic for the respective contexts and/or competing priorities of the MoH. Both countries that did not pilot IMCI activities were excluded from further analysis.

### Implementation of the three components of the IMCI strategy

#### Health worker training at PHC facilities and first-level hospitals

Fourteen countries piloted health worker trainings on the IMCI algorithm at PHC level and follow-up after-training activities in two to three districts. Eight of the countries scaled up trainings to nationwide coverage. Subsequently, 12 countries introduced the extension of IMCI to first referral level in the form of evidence-based guidelines, namely the *WHO Pocket Book of hospital care for children*, to promote quality care also at this level. Assessments of the quality of care at hospitals and trainings on the WHO Pocket Book were carried out in 11 countries.

#### Health systems

The second IMCI component aims to strengthen the health system for delivering quality child health services by adopting conducive policies, inter alia policies that make IMCI drugs available to children without cost to parents or caregivers. The inclusion of IMCI drugs in the National Essential Drug list was reported by 12 countries. Consistent availability of IMCI drugs free of charge for children was reported by key informants in only four countries. One country reportedly established a mechanism for supportive supervision, two countries had addressed inconsistencies between the IMCI classification and the International Classification of Diseases 10 (ICD 10) and two countries resolved incompatibilities between IMCI and other existing policies.

#### Family and community health practices

To improve parental health literacy, behaviour and care seeking, activities in the context of the community component were implemented in 11 countries. They included the integration of IMCI messages on key family practices into home visits in 8 countries, mass media campaigns in 9 countries and the distribution of education, information and communication materials for parents in 11 countries.

### Mortality reduction

In the eight countries in which IMCI was implemented at national scale, under-5 mortality rates were reduced, and key informants alleged unanimously that IMCI was likely to have contributed to this reduction, particularly in relation to deaths from pneumonia and diarrhoea. IMCI was also perceived as having contributed to the decrease in child mortality taking place at home.

In the seven countries where IMCI implementation was limited or non-existent, that is, where crediting IMCI with mortality reduction at a national level would not be plausible, under-5 mortality rates were also reduced.

No mortality data or estimates are available for the territory of Kosovo.

### Improved quality of care

Key informants from all participating countries reported that IMCI implementation promoted the use of evidence-based standard treatment guidelines and improved the quality of care. The use of IMCI algorithms by healthcare providers improved treatment decision-making through increasing the use of evidence-based clinical signs and symptoms to differentiate between children who would benefit from antibiotic or other prescriptions and those who would not; those who require hospitalisation and those who can be safely managed as outpatients. According to key informants, this resulted in a reduction of antibiotic misuse in 14 countries, of polypharmacy in 11 countries and of unnecessary hospitalisations also in 11 countries. In countries and territories with rural areas where primary healthcare is provided exclusively by nurses or feldshers, IMCI was praised for supporting systematic identification of danger signs and children in need of referral.

### Parents as partners

According to key informants, IMCI also contributed to improved breast feeding (in five countries), increased parental knowledge, particularly on the subjects of nutrition, danger signs and immunisation (in four countries) as well as improved care seeking as a determinant for reducing home deaths (in six countries).

### IMCI did not achieve maximum potential

**Figure 1:**
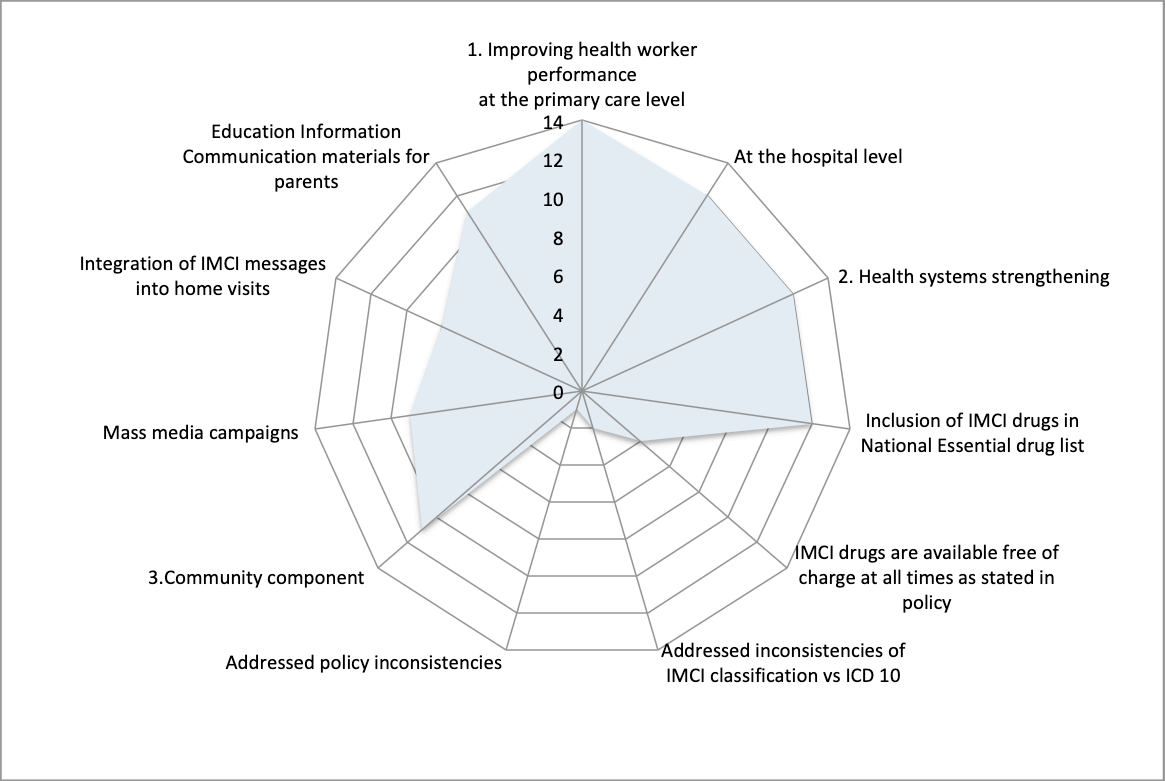
Implementation of the Integrated Management of Childhood Illness (IMCI) components 1–3 by number of countries (n=14). ICD, International Classification of Diseases.

The three components of IMCI were introduced unevenly in all countries with a strong focus on training for primary care health workers. The training of hospital (referral) care providers, the health system and community components lagged behind (figure 1). Implementation of each single component was often fragmented and not at national scale. Of the eight countries that scaled up IMCI training of primary care providers to national scale, five implemented the community component also at national scale.

Incompatibilities between IMCI and existing policy requirements and regulations were not addressed consistently, hindering IMCI implementation: examples include inconsistencies between ICD 10 and IMCI classifications (reported to have caused difficulties in 14 countries, but only addressed in 2), policy requirements to admit children with diarrhoea to infectious diseases hospitals and persisting practices for investigating stool samples in former Soviet Union countries (reported by key informants in 6 countries).

Key informants in 11 countries, particularly from academia and professional organisations, perceived the IMCI approach as dogmatic: the exclusion of diagnostic tools widely available in the region from the algorithm, such as urine dipsticks, haemoglobin blood test and particularly the de facto ban of stethoscopes discredited IMCI with academia and professional organisations.

### IMCI achievements made were not sustained

At the time of the review almost two decades after its introduction, IMCI activities were still ongoing in eight countries. Criteria considered to assess sustainability included (1) the presence of a legislative base for IMCI implementation, (2) the integration of IMCI training into preservice training and (3) the integration into continuous education.

All three criteria were present concomitantly in four countries and thus IMCI implementation was considered sustainable. In six countries, key informants reported that IMCI activities stalled or stopped completely when external support ceased. In the remaining countries, only one of the criteria was reported as present. Ukraine was excluded from the analysis for sustainability, as IMCI implementation had started only recently and no conclusion could be drawn on whether it will be sustainable in the long term.

Key informants from 14 countries reported that improvements in the quality of care, namely reduced misuse of antibiotics, reduced polypharmacy and reduced rates of unnecessary hospitalisation were not sustained over time. Reasons cited to explain this regression included the lack of continuous follow-up after training, lack of supportive supervision and offsetting health system constraints.

### System issues affecting the problems IMCI is trying to address

The consideration of aspects other than evidence and the best interest of the child in treatment decisions such as economic factors were reported by 13 countries, where under-resourcing and the disintegration of health systems had perpetuated the development of commercialised healthcare (defined here as the unregulated fee-for-service sale of healthcare, regardless of whether or not it was supplied by public or private providers) (box 1).

Box 1 **Commercialisation of medicine**

**Key informants’ comments on misaligned monetary and health goals.**

Treatment and hospitalisation of children (is) funding driven; hospitals are forced to keep children up to 10 days for treatment of pneumonia to receive full payment.

Many jobs depend on high hospitalisation rates and overtreatment and overmedicalisations. (Implementation) of Integrated Management of Childhood Illness (IMCI) would lead to job losses.

Continuous medical education in place, but preference given to professors with unclear commercial ties who might advertise specific brands, no mechanisms (exist) to keep commercial interests at bay.

Paediatricians try to create a demand; more patients will lead to more treated cases and money.

IMCI is not implemented as payment for the patient is depending on hospitalisation, the hospital puts the outpatient into the hospital to get funding, because they need patients otherwise the hospital will be shut down.

In the hospital the doctor earns money based on the number of patients.

There is aggressive marketing of baby food in the delivery hospitals and doctors have an interest to promote formula.

The health sector has become a money-making system.

Why focusing on IMCI when you need more patients, more exams and IMCI teaches using less. It is not profitable.

Nobody pays for consultation alone, so it is in the interest of the paediatrician to attach many diagnoses to a child and see the child often, creating a ‘chronically sick child’. For example, a normal hormone-induced rash in newborns will be classified as atopic eczema, leading to a numerous laboratory tests (lactose, streptococci in breast milk, etc), follow-up visits, treatments, advice on food to avoid.

Key informants from 11 countries reported that parents perceived the quality of care provided at PHC level as poor, particularly in the case of curative health services. According to key informants, this perception along with a preference among parents for medicalised care (ie, preferring invasive or active treatment over conservative or watchful management, intravenous treatment over oral rehydration therapy and multiple drugs over just one), led to parents often bypassing primary care: taking their children directly to specialists at secondary or tertiary care level (if accessible) and seeking more sophisticated diagnostic tests and treatments. PHC facilities were often only used for preventive measures, such as immunisations and healthy child monitoring visits.

In 11 countries key informants reported that out-of-pocket expenditure by parents or guardians was required despite existing conducive policies (box 2).

Box 2 **It is for free, unless it is not**

**Key informants’ comments on the implementation of the policy to make drugs and services available to children free of charge.**

The Integrated Management of Childhood Illness (IMCI) drugs are included in the National Drug List, however, drugs are not available. Eighty-five per cent of drugs are paid for out-of-pocket.

Essential IMCI drugs were available but people had to pay for drugs—and still have to.

Services for children under three are for free but drugs are not.

Immunisation and services are for free but drugs are not. IMCI drugs are relatively cheap compared with other antibiotics but still too expensive…(people) will buy some tablets but not the whole prescription.

Medications such as antibiotics need to be paid by the patients themselves in primary care, however, this treatment is free in hospitals.

Drugs are included into (the) National Essential Drug list, and drugs for emergency care are available at health facilities, including prehospital treatment. Drugs are not free for the full course. Parents have to purchase prescribed medicines.

Funds are limited and drugs are procured by parents.

The IMCI programme assures free drugs. (Over) time the list of IMCI drugs was reduced from 13 to 3.

IMCI drugs were included in the National Essential Drug list, but not all of the medicines were available in health facilities. Primary healthcare facilities’ funding for IMCI medicines is limited, and frequently parents had to purchase drugs.

Officially, there was no cost for patients, and medicines were provided through international agencies, but as informal payments in healthcare are common in the country, it is hard to say for certain.

Reasons cited included the prescription of drugs not belonging to the group of IMCI drugs either because they were believed to be superior (both by doctors and parents), or due to revenue generation and/or kickbacks through pharmaceutical companies. Details on the improved rational use of drugs particularly antibiotics for children and the mechanisms through which these were achieved as well as counteracting system factors, are discussed elsewhere.^9^

Key informants in four countries reported that unnecessary and prolonged hospitalisation is incentivised by financing mechanisms and payment schemes of healthcare services: reimbursements for hospitals and providers (supply side) depend on hospitalisation rates and are superior for services provided to inpatients and linked to hospitalisation for a minimum number of days. Incentives for the patient (demand side) include the availability of drugs, diagnostic and treatment services free-of-charge at the hospital, while to be covered out-of-pocket in case of outpatient treatment.

Working conditions that are likely to have a negative impact on primary healthcare workers’ ability and motivation to adhere to evidence-based standard treatment guidelines were reported by key informants from all countries and included one or more of the following: low salaries, influence from pharmaceutical and/or formula milk producing industries, dilapidated and unreliable infrastructure (eg, intermittent or no supply of warm water, heating and/or electricity) in some primary healthcare facilities and no access to continuing medical education or peer support.

## Discussion

The findings of our review align with the Cochrane review and global IMCI review in relation to IMCI’s wide acceptance as a sound evidence-based approach to child health.^2 6 10–13^ Our review also reiterates the widely described limitations created by inefficient and fragmented health systems on IMCI implementation at national scale with adequate quality.^14–16^ To our knowledge, this is the first attempt to investigate to what extent these health system limitations have been addressed in order to enable IMCI implementation. It also externalises inherent incentives and underlying motivational factors that may explain why IMCI has never been fully used either as a strategy or as an algorithmic diagnostic and treatment decision tool despite being highly regarded by service providers and policy makers.^6^

### Limitations

A weakness of the review was that it relied on interviews and focus groups with key informants and reviewers, many of which had an intellectual interest in IMCI, as they had been involved in its development and implementation, hence introducing bias. However, end-users of the IMCI algorithms lacking such bias were also interviewed. The need for translation during some key informant interviews and the fact that respondents may not feel comfortable providing answers that present a WHO/UNICEF strategy and/or the country in an unfavourable manner may have contributed to inaccurate answers.

To limit bias and expose inaccurate answers, the iterative questioning technique was used; statements were reviewed together with the respondent for accuracy taking into account forms of interaction, power relationships and established interpretations of the IMCI approach. Particular care was taken to disentangle policy from what was happening in reality; to differentiate between what was considered to be an effective approach on a theoretical basis and what was observed to have worked well.

Further limitations are mostly due to the qualitative review design; however, it highlights important factors limiting the achievement of IMCI’s full potential and allowed us to explore how the systems affect the problems IMCI is trying to address. Through triangulation of data collected through desk reviews, individual and focus group interviews, findings were cross-validated, and some of the inherent weaknesses in qualitative studies overcome, adding important information to the evidence base.

### Conflict between economic and health goals

Our review showed that IMCI implementation did not sufficiently address the following issues in primary healthcare for children that—in contravention to the obligations under the Convention on the Rights of the Child^17^—persist in the European region:

- Non-evidence-based practices, particularly the indiscriminate use of antibiotics and polypharmacy.
- Inappropriate medicalisation with diagnostic and treatment approaches without therapeutic value.
- Unnecessary and prolonged hospitalisation.
- Lack of health promotion particularly support for breast feeding, and healthy growth and development for children.

In addition, our review found that despite the widespread and long-standing recognition of supportive health systems being necessary for effective IMCI implementation, efforts to address health systems barriers to IMCI implementation were limited.^13–15^

Most if not all of these practices and the potential disinterest in addressing the well-known health systems constraints might be explained by conflicts between economic and health goals. The provider in some situations had an interest in investigating and prescribing what is most profitable and not necessarily what is best for the child. This is likely to be amplified by low salaries and governments that fail to regulate the health sector either by choice or due to a lack of capacity to do so.

As elsewhere in the world, IMCI implementation in the European region focused on health-worker training, which by itself is unlikely to lead to performance improvement. Under the best of circumstances it may lead to improved knowledge and skills, whether or not these are applied in real life for the benefit of children depends on a myriad of enabling system factors as well as on personal motivation.^18^ Insufficient preparation during preservice education cannot be expected to be remedied by a 10-month retraining, let alone a 10-day training course in IMCI.^19^ Integration into preservice training and continuous professional education was achieved only in a limited number of countries and with varying quality. As a result, parents gravitated to specialised and often commercialised care providers, which they perceived to be more competent.

### IMCI in the context of primary healthcare and universal health coverage

Four decades after the Alma-Ata Declaration, its core values and principles seem—in the zeitgeist of universal health coverage (UHC) and people-centred health systems—as pertinent and modern as ever.^20–22^ The goal of UHC is defined as ‘ensuring that all people have access to promotive, preventive, curative and rehabilitative health services, of sufficient quality to be effective, while also ensuring that people do not suffer financial hardship when paying for these services’.^23^ For children UHC will remain elusive, if underlying system pressures are not addressed. The continued focus on disease-specific approaches combined with the priority shift towards non-communicable diseases threatens to wipe out achievements made in child health. Making healthcare truly universal for children will require a shift in a different direction: towards health systems designed around and for children away from systems centred on providers’ needs and parents’ expectations. Health systems need to produce health workers sufficient in number, trained in evidence-based practices before deployment and able to access continuous medical education thereafter. It must enable its health workers to base treatment decisions on what is best for the child and not what might be the most lucrative, quickest fix or safest bet; prerequisites of which will be sufficient remuneration, sound training, improved parental health literacy, conducive laws and regulations and sound reimbursement systems with adequate checks and balances to ensure the best possible care.^24^ Innovative tools and mechanisms to support implementation of standard treatment guidelines, such as collaborative approaches and the use of new technologies should be used to full capacity.^25 26^

## Conclusion

The renewed Alma-Ata Declaration discussed last October in Almaty, Kazakhstan has reconfirmed the emphasis on primary care as the main driver for achieving universal health coverage for children.^13^ Standardised and evidence-based treatment approaches, built on the IMCI guidelines, will be required as the basis for the definition of standards of care, to be implemented by a sufficiently remunerated work force with the necessary competencies. Work in the following three areas must underpin any serious notion of universal health coverage for children:

1. Updating guidelines for care for children, including newborns and adolescents, to be delivered at PHC level based on evidence and taking into account the child’s rights as the basis for defining standards of care.
2. Strengthening pregraduate and postgraduate education for primary care providers.
3. Ensuring that health systems and laws and regulations are responsive to the needs of children, including adequate checks and balances to ensure care provision is based on evidence and in the best interest of the child.

Governments must honour their responsibilities as enshrined in the Convention on the Rights of the Child to provide adequate care for all children.^13^

Most of all, we, the global health community, will have to disenthrall ourselves from the notion that healthcare is a commodity and can be left to market forces, to ensure provision based on evidence with quality and equity.
